# The ryanodine receptor mutational characteristics and its indication for cancer prognosis

**DOI:** 10.1038/s41598-022-19905-y

**Published:** 2022-09-27

**Authors:** Fenglin Wang, Jingbo Yu, Ping Lin, Charalampos Sigalas, Shibo Zhang, Yuan Gong, Rebecca Sitsapesan, Lele Song

**Affiliations:** 1 College of Life Sciences, Nankai University, Tianjin, 300071 People’s Republic of China; 2 Department of Pharmacology, University of Oxford, Mansfield Road, Oxford, OX1 3QT UK; 3 Department of Radiotherapy, The Eighth Medical Center of the Chinese PLA General Hospital, Beijing, 100091 People’s Republic of China; 4 Department of Hepatobiliary Surgery, Dalian Municipal Central Hospital, Dalian Medical University, Dalian, 116033 Liaoning Province People’s Republic of China; 5 Department of Ultrasound, The First Affiliated Hospital of Dalian Medical University, Dalian, 116011 Liaoning Province People’s Republic of China; 6 Department of Thoracic Surgery, Harbin Medical University Cancer Hospital, Harbin, 150081 Heilongjiang Province People’s Republic of China; 7 Department of Gastroenterology, The Second Medical Center of the Chinese PLA General Hospital, Beijing, 100853 People’s Republic of China

**Keywords:** Cancer genomics, Genetic markers

## Abstract

Ca^2+^ signaling is altered substantially in many cancers. The ryanodine receptors (RYRs) are among the key ion channels in Ca^2+^ signaling. This study aimed to establish the mutational profile of RYR in cancers and investigate the correlation between RYR alterations and cancer phenotypes. The somatic mutation and clinical data of 11,000 cancer patients across 33 cancer types was downloaded from The Cancer Genome Atlas (TCGA) database. Subsequent data processing was performed with corresponding packages of the R software. Mutational profile was analyzed and its correlation with tumor mutational burden (TMB), patient prognosis, age and smoking status was analyzed and compared. All three RYR isoforms exhibited random mutational distribution without hotspot mutations when all cancers were analyzed together. The number of mutations in RYR2 (2388 mutations) far overweight that of RYR1 (1439 mutations) and RYR3 (1573 mutations). Linear correlation was observed between cumulative TMB and cumulative number of mutations for all RYR isoforms. Patients with RYR mutations exhibited significantly higher TMB than those without RYR mutations for most cancer types. Strong correlation was also revealed in the average number of mutations per person between pairs of RYR isoforms. No stratification of patient overall survival (OS) by mutational status was found for all three RYR isoforms when all cancers were analyzed together, however, significant stratification of OS by RYR mutations was revealed in several individual cancers, most strikingly in LUAD (P = 0.0067, RYR1), BLCA (P = 0.00071, RYR2), LUSC (P = 0.036, RYR2) and KIRC (P = 0.0042, RYR3). Furthermore, RYR mutations were correlated with higher age, higher smoking history grading and higher number of pack years. Characteristic mutation profile of RYRs in cancers has been revealed for the first time. RYR mutations were correlated with TMB, age, smoking status and capable of stratifying the prognosis of patients in several cancer types.

## Introduction

Ca^2+^ signaling is important physiologically and pathologically. Ca^2+^ functions as a second messenger and is involved in the regulation of nerve conduction, muscle contraction, morphological changes, cell differentiation, gland secretion and other physiological processes^1^. Under pathological conditions, Ca^2+^ signaling can be activated and involved in inflammatory responses, regulation of protein and kinase activity, cytoskeletal remodeling, regulation of extracellular matrix, apoptosis and other processes^1^. The intracellular Ca^2+^ is regulated through various Ca^2+^ pumps and Ca^2+^ channels. The former include sarcoplasmic/endoplasmic (SR/ER) reticulum Ca^2+^ ATPase (SERCA), plasma membrane Ca^2+^ ATPase (PMCA) and sodium-Ca^2+^ exchanger. The latter mainly includes voltage-gated Ca^2+^ channel (VGCC), ligand-gated Ca^2+^ channel (LGCC) and transient receptor potential (TRP) ion channels^2^. VGCC includes 10 types of channels^3^ and LGCC includes SR/ER Ca^2+^ channels, including inositol trisphosphate receptor (IP3R) and ryanodine receptor (RYR)^1^.

Ca^2+^ signaling abnormality is one of the common alterations in tumors^1^. Ca^2+^ signaling may be remodeled in cancer, and its remodeling may influence key events such as proliferation, invasion and sensitivity to cell death in cancer^1^. Specific Ca^2+^ signaling pathways have also been shown to play important roles in the establishment and maintenance of multidrug resistance and new microenvironment^1^. RYR is a type of Ca^2+^ release channel that can quickly release Ca^2+^ from SR/ER, thereby increasing intracellular Ca^2+^ concentration, stimulating further Ca^2+^ activation, and playing important roles in activating Ca^2+^-activated potassium channels, excitation–contraction coupling and other physiological functions^4^. The activity of RYR is regulated by many ions or small molecules (such as Ca^2+^, magnesium, caffeine and ATP) and by many closely associated proteins (such as calmodulin and FK506 binding protein)^4^. There are three subtypes of RYR, including RYR1 (mainly in skeletal muscle), RYR2 (mainly in heart muscle) and RYR3 (more widely distributed, mainly in the brain). Although the role of RYR has been extensively studied in skeletal, cardiac, and neurological diseases, its exact role in cancer transformation and development has rarely been studied. More specifically, the mutational landscape of the three RYR isoforms and the roles of their mutations in various types of cancers have not been systematically studied so far. Since RYRs are large proteins involved in key steps of Ca^2+^ signaling, it is worth investigating their roles in cancers.

Here we performed the first comprehensive study of RYR mutational landscape and its correlation with cancer phenotypes regarding all recorded cancer types from the TCGA database. We identified characteristic RYR mutational status in cancers and established its correlation with TMB, patient prognosis, age and smoking status. Our study provided the first insightful observation on RYR genetic alterations and their potential influences in cancer.

## Methods and materials

The somatic mutation data along with demographic and clinical information of 11,000 patients across 33 cancer types was downloaded from The Cancer Genome Atlas (TCGA) database (https://portal.gdc.cancer.gov/) in Mutation Annotation Format (MAF) format using the “TCGAbiolinks” package in R software (https://www.rstudio.com/) on April 20th, 2021. The demographic and clinicopathological information for all patients was summarized in Table 1. Mutation profile and tumor mutational burden (TMB) were analyzed with the “maftools” package. The distribution of RYR1, RYR2, RYR3 mutations was displayed by lollipop plot and histogram with bins at 150 bases (50 amino acids) using the “maftools” of R software.

**Table 1:** Demographic and clinicopathological information for subjects involved in this study.

Factors	Categories	Number of subjects
Sex	Male	5397
	Female	5603
Age	< 40	868
	40–49	1192
	50–59	2214
	60–69	2693
	70–79	1951
	≥ 80	685
	Not specified	1397
Smoking history*	1	868
	2	759
	3	661
	4	877
	5	61
	Not specified	7774
Cancer types	ACC	98
	BLCA	573
	BRCA	1054
	CESC	297
	CHOL	58
	COAD	446
	DLBC	37
	ESCA	199
	GBM	390
	HNSC	541
	KICH	73
	KIRC	389
	KIRP	338
	LAML	134
	LGG	520
	LIHC	400
	LUAD	603
	LUSC	555
	MESO	88
	OV	438
	PAAD	176
	PCPG	209
	PRAD	515
	READ	146
	SARC	278
	SKCM	490
	STAD	447
	TGCT	133
	THCA	518
	THYM	133
	UCEC	575
	UCS	65
	UVM	84
Clinical stages	I	2138
	II	2251
	III	1763
	IV	888
	Not specified	3960
RYR1 mutational status	WT	10,018
	Mutant	982
RYR2 mutational status	WT	9508
	Mutant	1492
RYR3 mutational status	WT	9976
	Mutant	1024

*1 = Lifelong Non-smoker (less than 100 cigarettes smoked in Lifetime), 2 = Current smoker (includes daily smokers and non-daily smokers or occasional smokers), 3 = Current reformed smoker for > 15 years (greater than 15 years), 4 = Current reformed smoker for ≤ 15 years (less than or equal to 15 years), 5 = Current reformed smoker, duration not specified.

All patients were divided into mutation group (Mut) and wide type group (WT) in mutation status analysis for RYR1, RYR2 and RYR3 (Table 1). Wilcoxon test was performed to compare the continuous random variables between the Mut and the WT groups, including TMB, number-pack-years-smoked, tobacco-smoking-history, age-at-initial-pathologic-diagnosis. Linear regression was performed to analyze the correlation between cumulative number of mutations and cumulative TMB and the average number of mutations between RYR isoform pairs. Kaplan–Meier analysis and log-rank test were performed to investigate the potential stratification of RYR1, RYR2, RYR3 mutations on patient overall survival in all cancers and individual cancers. *P < 0.05; **P < 0.01; ***P < 0.001.

## Results

### The mutational characteristics of RYRs in cancers

**Figure 1:**
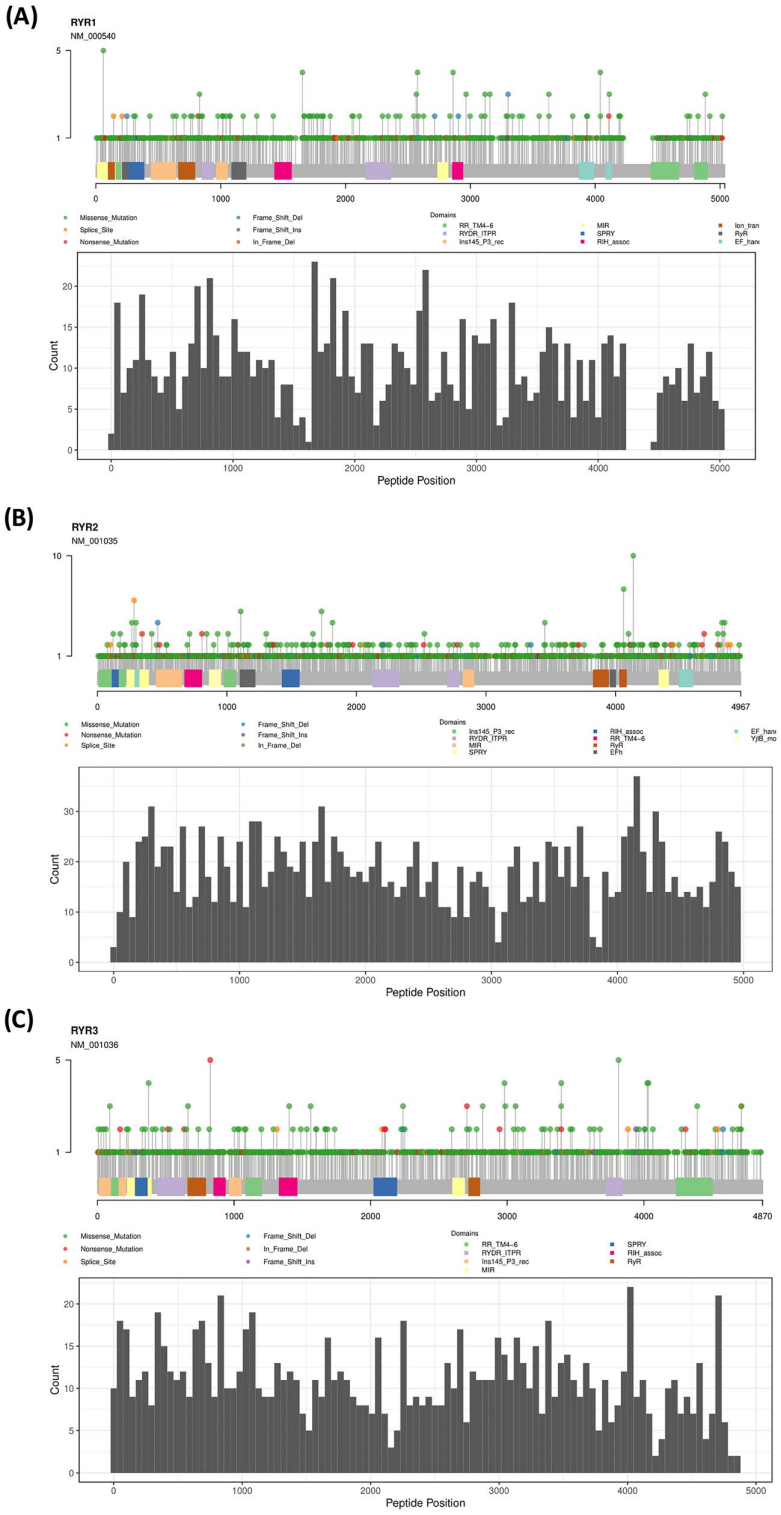
The mutational landscape for RYR1,RYR2 and RYR3 in cancer patients. All mutations identified in TCGA database are plotted on the schemes of RYR1 (**A**), RYR2 (**B**) and RYR3 (**C**). Mutations are presented by dots, and colors represent mutation types. The Y-axis represents the number of mutations for each site. Key RYR domains are labeled as indicated. The histograms in (**A**–**C**) show the number of mutations of RYR1,RYR2 and RYR3 for every 150 bases (50 amino acids), representing the frequency of mutations at 150-base frame.

To study the mutation profile of RYR in various types of cancers, we first investigated the distribution of mutations by mapping all RYR1, RYR2 and RYR3 mutations from 10,114 cancer patients recorded in the TCGA database (Fig. 1). A total of 1439 RYR1 mutations were mapped in Fig. 1A upper panel. It can be seen that mutations were evenly distributed throughout the whole length RYR1. Most mutations appeared only once, while some mutations appeared several times, with the most frequent mutation occurring 5 times. We calculated the number of mutations of RYR1 per 150 base pairs (bp) (50 amino acids) and plotted the mutation frequency of RYR1 (Fig. 1A, Lower Panel). It can be seen that the mutation frequency varied greatly across the 150 bp frames indicating that there were no clear hotspot mutations or hotspot mutational regions in cancers in RYR1, and that the mutation distribution may be random. Figure 1B and C show the mapping of mutations in RYR2 and RYR3, respectively. It was found that RYR2 (2388 mutations) had much higher number of mutations than RYR1 (1439 mutations) and RYR3 (1573 mutations). Similar to RYR1, most RYR2 and RYR3 mutations occurred once, while some occurred more than once. The distribution showed a fluctuation without clear hot spot mutations and hot spot mutational regions. In summary, RYR1, RYR2 and RYR3 showed similar random mutation distribution patterns, indicating no significant difference among the three isoforms.

**Figure 2:**
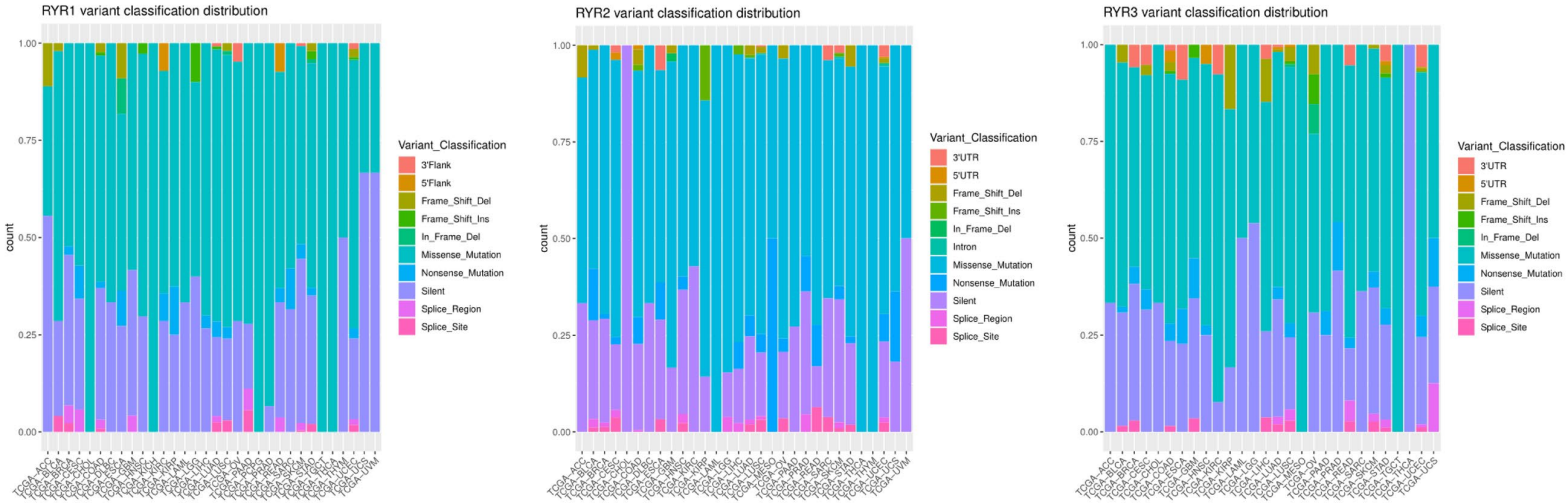
Distribution of mutational types in a series of cancer types in RYR1, RYR2 and RYR3. The left, middle and right panels represent the ratio of various types of mutations in RYR1, RYR2 and RYR3, respectively. The types of cancers are aligned as indicated. Colors represent different types of mutations, as indicated by the illustrations.

The mapping of mutation distribution in Fig. 1 included all cancer types. In order to understand the distinct mutational status of each cancer type, we further analyzed the ratio of RYR1, RYR2 and RYR3 mutation types in 30 different cancers. Although Fig. 2 shows a huge variation in the mutation types across the various cancers, the main types of mutations included missense mutations and silent mutations, with a small proportion of nonsense mutations, splicing mutations and frameshift mutations. Some cancers were dominated by a certain mutation type. For example, RYR1 in cholangiocarcinoma (CHOL) and RYR3 in testicular germ cell tumors (TGCT) only had missense mutations, and RYR2 in CHOL and RYR3 in thyroid carcinoma (THCA) only had silent mutations. This may be due to the bias resulting from low number of mutations and/or small sample size in these cancer types. These observations showed that most cancer types had a similar ratio of mutation types, exhibiting a relatively consistent mutational distribution across the three RYR isoforms.

**Figure 3:**
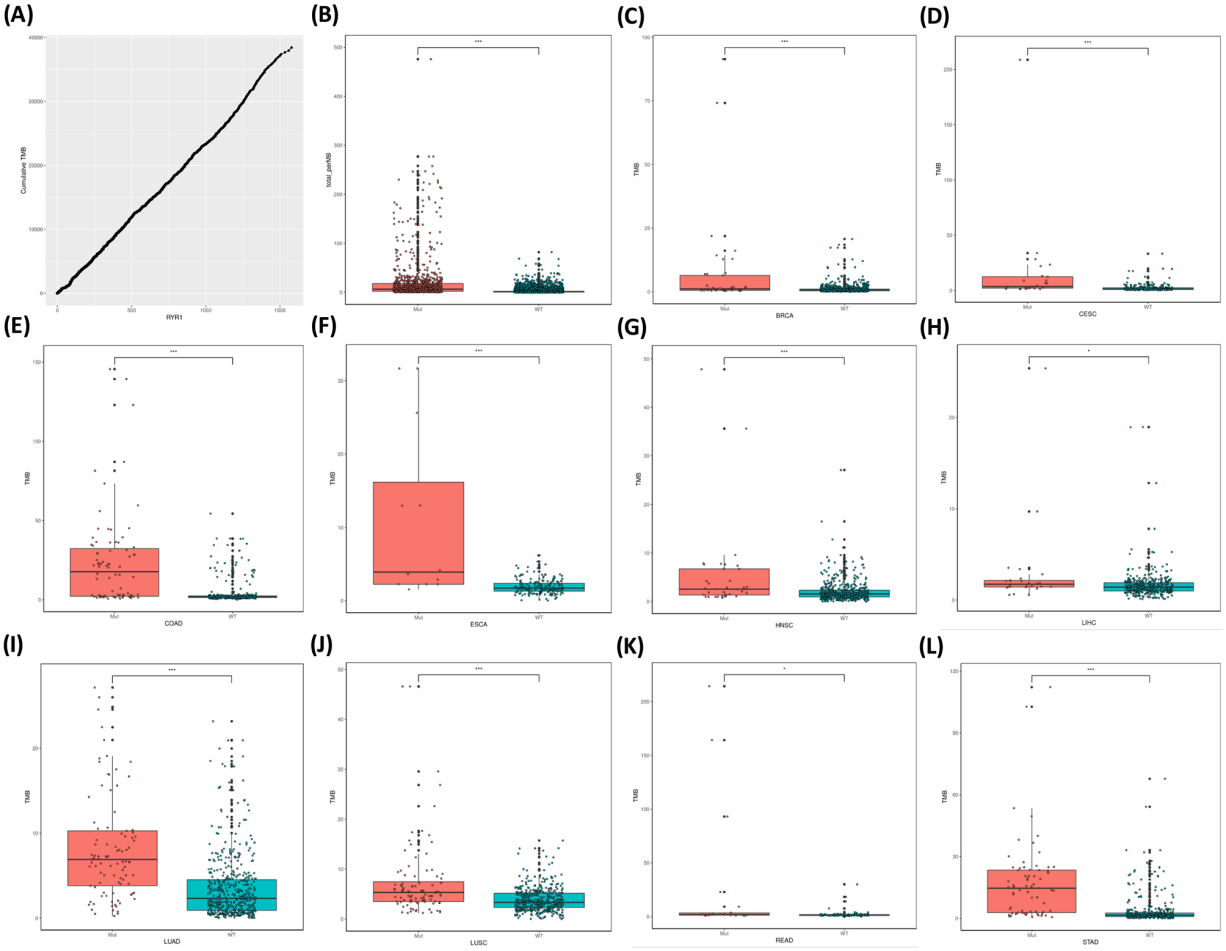
The correlation between RYR1 mutational status and tumor mutational burden (TMB). (**A**) the correlation between cumulative number of mutations (X-axis) and cumulative TMB (Y-axis) in RYR1. (**B**) scatter plot of TMB grouped by RYR1 mutational status (mutant or WT) in all types of cancers. (**C**–**L**) scatter plot of TMB grouped by RYR1 mutational status (mutant or WT) in each type of cancer, as indicated. *P < 0.05; **P < 0.01; ***P < 0.001.

**Figure 4:**
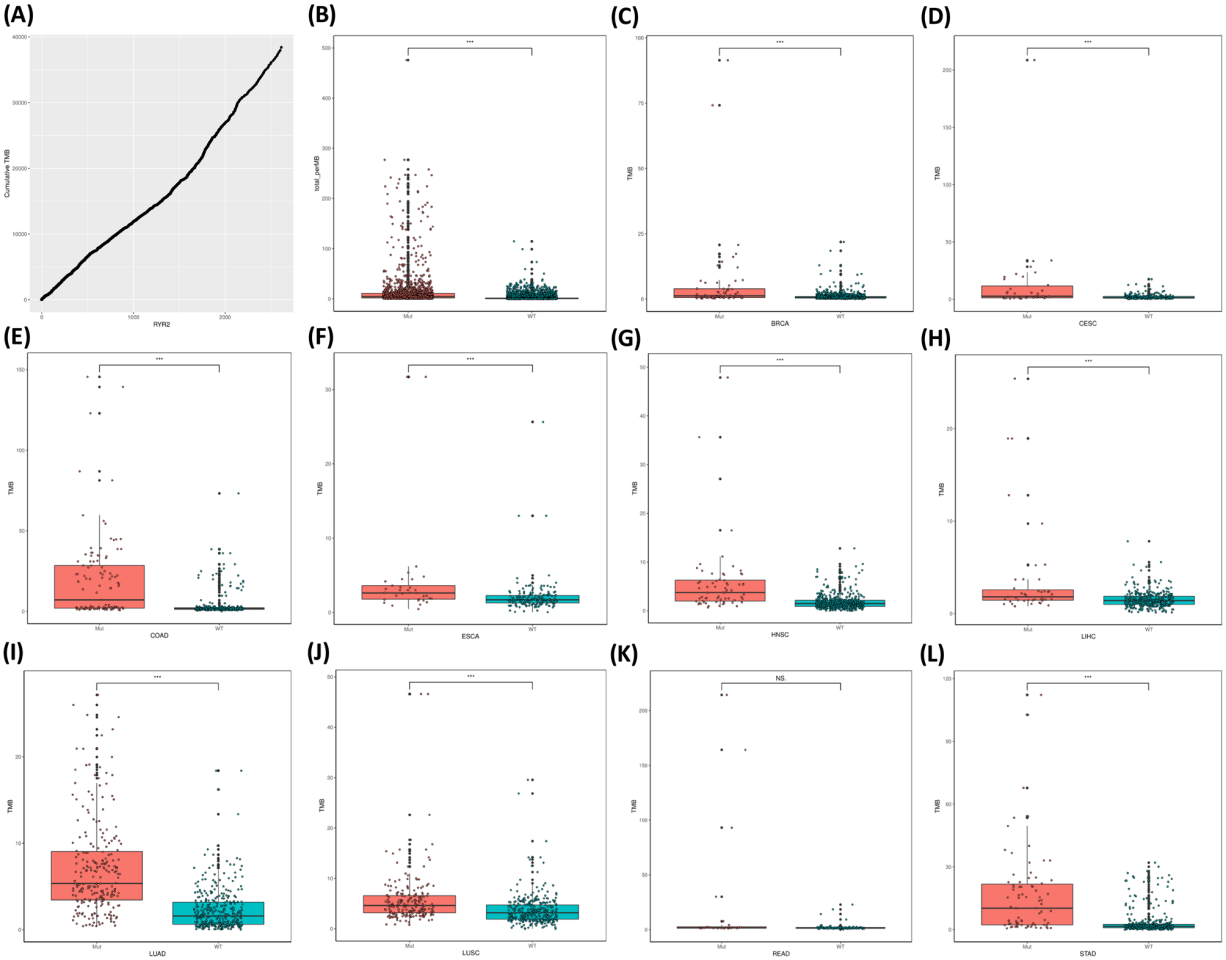
The correlation between RYR2 mutational status and tumor mutational burden (TMB). (**A**) The correlation between cumulative number of mutations (X-axis) and cumulative TMB (Y-axis) in RYR2. (**B**) Scatter plot of TMB grouped by RYR2 mutational status (mutant or WT) in all types of cancers. (**C**–**L**) Scatter plot of TMB grouped by RYR2 mutational status (mutant or WT) in each type of cancer, as indicated. *P < 0.05; **P < 0.01; ***P < 0.001.

**Figure 5:**
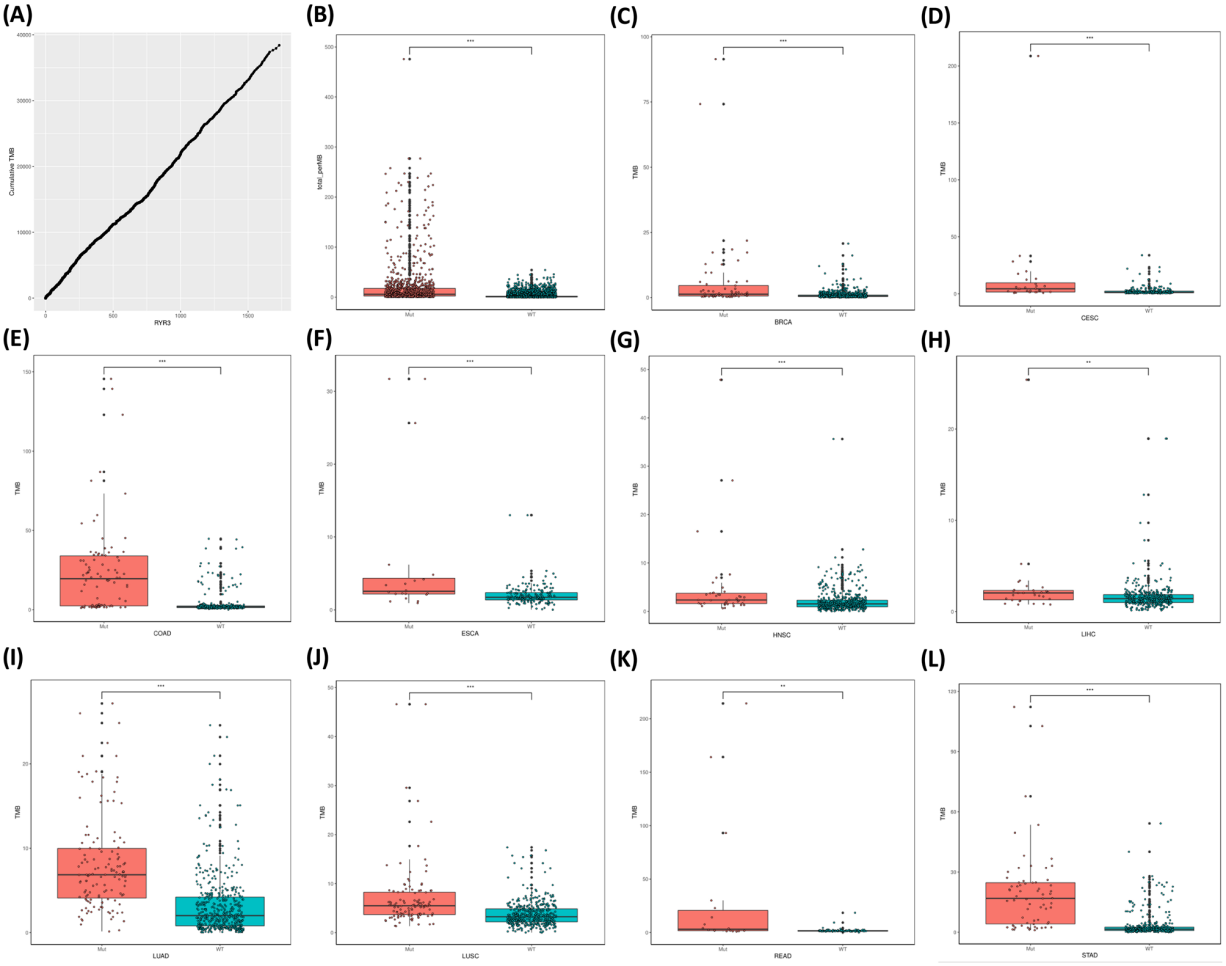
The correlation between RYR3 mutational status and tumor mutational burden (TMB). (**A**) The correlation between cumulative number of mutations (X-axis) and cumulative TMB (Y-axis) in RYR3. (**B**) Scatter plot of TMB grouped by RYR3 mutational status (mutant or WT) in all types of cancers. (**C**–**L**) Scatter plot of TMB grouped by RYR3 mutational status (mutant or WT) in each type of cancer, as indicated. *P < 0.05; **P < 0.01; ***P < 0.001.

Tumor mutational burden (TMB) is calculated as the number of mutations per million bases. Due to the limited number of RYR mutations in each cancer patient, it is not accurate to calculate the correlation between the number of RYR mutations and TMB for each patient. Therefore, we calculated the correlation between the cumulative number of mutations and the cumulative TMB. Figure 3A shows a very clear linear correlation between the RYR1 cumulative number of mutations and the cumulative TMB. Figure 3B shows that patients with RYR1 mutations had significantly higher TMB than those without RYR1 mutations (P < 0.001). The same trend can be found in all representative cancer types as indicated in each panel (P < 0.05, Fig. 3C–L). Similarly, Figs. 4A and 5A also show a very clear linear correlation between the cumulative number of mutations and the cumulative TMB in both RYR2 and RYR3, respectively. Figures 4B and 5B also showed that patients with either RYR2 or RYR3 mutations had significantly higher TMB than patients without mutations (P < 0.001). This pattern was also present in almost all individual cancer types (P < 0.01, Fig. 4C–J, except Fig. 4K (not significant, NS), Fig. 4L, and Fig. 5C–L,). The above studies indicate that there was a significant linear correlation between the number of RYR mutations and TMB.

**Figure 6:**
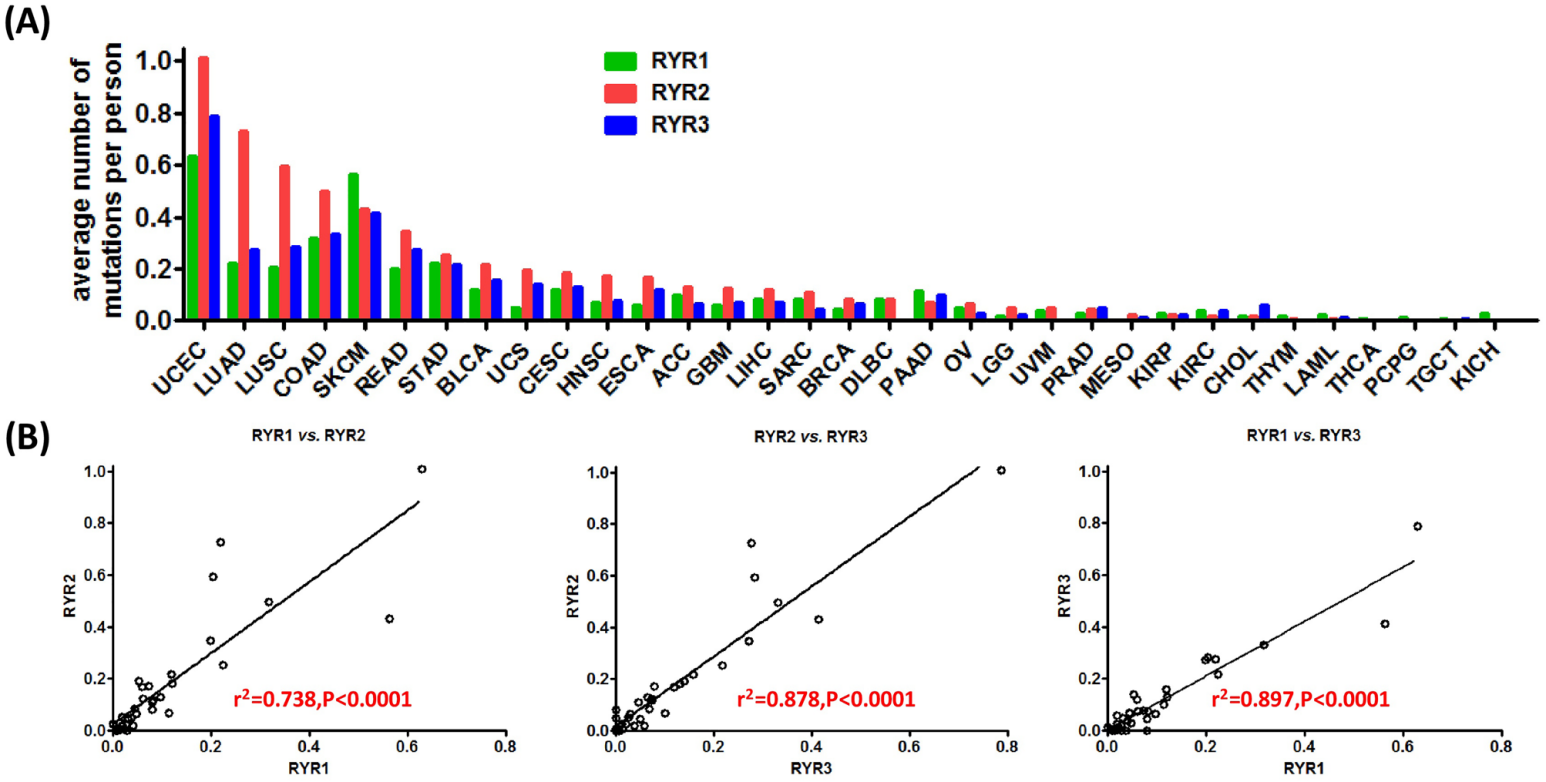
Comparison of average number of mutations and correlation of average number of mutations between RYR subtypes. Top panel: the average number of mutations per person of RYR1, RYR2 and RYR3 is compared for each cancer type, ranked by the average number of mutations of RYR2. Bottom panel: correlation of average number of mutations between any two RYR subtypes for all cancer types. Correlation coefficient (r^2^) and P values are labeled for each correlation.

To understand the similarities and differences of cancer mutational status across RYR1, RYR2 and RYR3, we further analyzed the average number of mutations of the three RYR isoforms in different cancer types. Figure 6A shows the average number of mutations per person, ranked by RYR2 average mutation numbers. It can be seen that the average number of mutations varied greatly in different cancer types, and uterine corpus endometrial carcinoma (UCEC), lung adenocarcinoma (LUAD), lung squamous cell carcinoma (LUSC), skin cutaneous melanoma (SKCM) and rectum adenocarcinoma (READ) were those with highest average number of mutations. The average number of RyR2 mutations per person was generally higher than that for RYR1 and RYR3, mostly strikingly in LUAD, LUSC and colon adenocarcinoma (COAD). Figure 6B show the correlation of the average number of mutations per person between any two of the three RYR isoforms. Strong linear correlation was observed between RYR1 and RYR2 (r^2^ = 0.738, P < 0.0001), between RYR2 and RYR3 (r^2^ = 0.878, P < 0.0001) and between RYR1 and RYR3 (r^2^ = 0.897, P < 0.0001). These observations indicate that the average number of mutations per person among RYR isoforms was highly correlated, suggesting a consistent trend across different cancer types.

In order to further investigate whether the above correlation was unique to RYRs, we established the mutational frequency of major proteins in calcium signaling. Supplementary Fig. 1 shows the top 15 mutated genes in calcium signaling in six representative cancers (LUAD, LUSC, COAD, READ, STAD and UCEC). It can be seen that RYRs were always the top 3 mutated genes, and RYR2 was always the top mutated genes in the six cancers. Interestingly, other large proteins of the calcium signaling pathway, including several types of voltage-gated calcium channel alpha1 subunit (CACNA1) and inositol 1,4,5-trisphosphate receptor (ITPR), were also among the top 15 mutated genes. This observation suggests that calcium channels, especially RYRs, were predominantly affected in cancers. The correlation between RYRs and TMB could also be present in other calcium channels.

### Stratification of cancer prognosis by RYR mutational status

**Figure 7:**
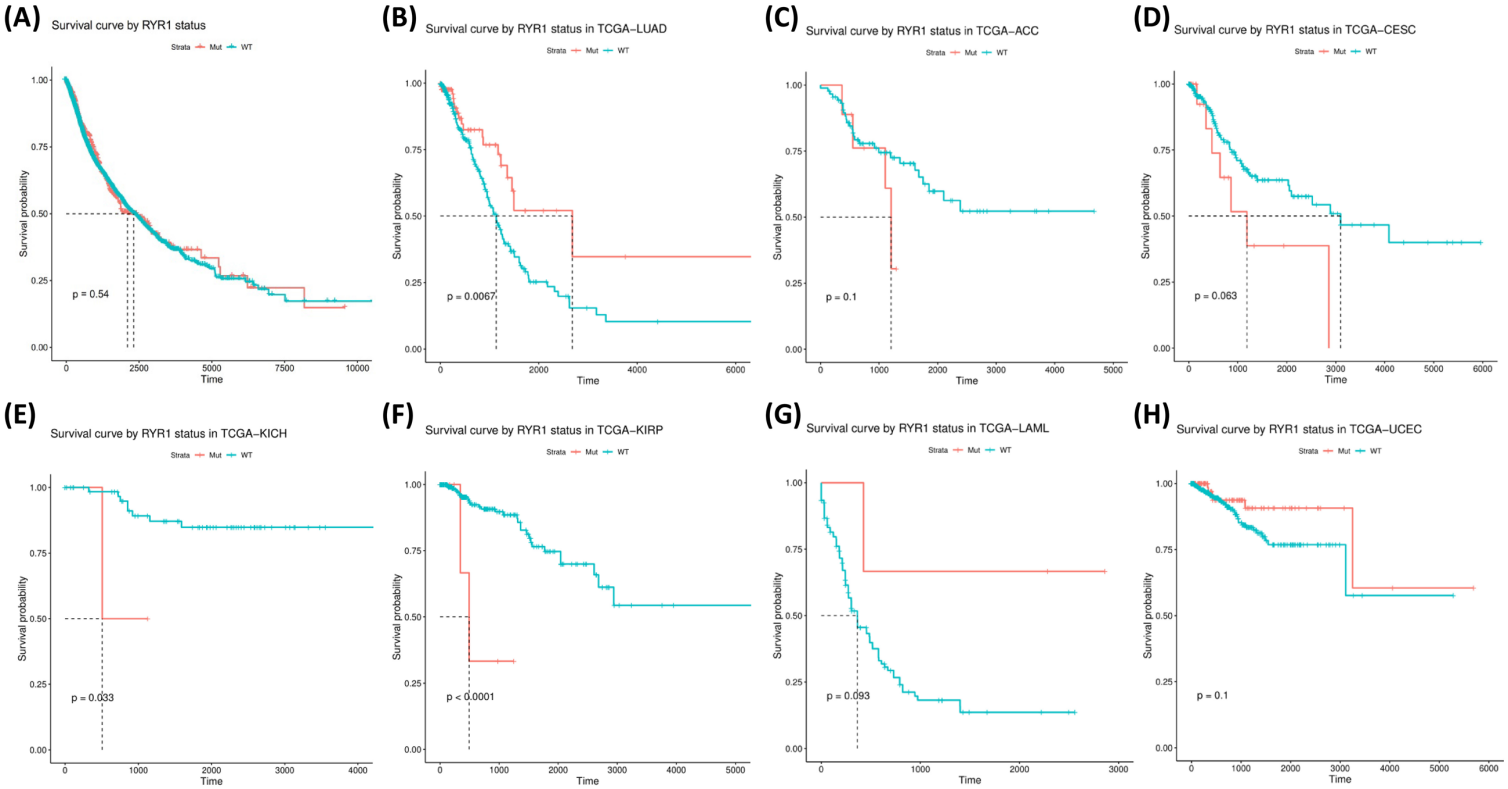
Kaplan–Meier survival analysis based on RYR1 mutational status (mutant or WT). The data for overall survival (OS) time is shown for each subgroup. (**A**) The survival analysis based on RYR1 mutational status for all types of cancers. (**B–H**) The survival analysis based on RYR1 mutational status for each type of cancer, as indicated. Only cancer types with P ≤ 0.1 are shown. P values are indicated in each panel. The unit for time is days.

Kaplan–Meier survival analysis was performed to investigate the potential stratification of patient prognosis by RYR mutational status (with or without RYR mutations). Figure 7 shows the stratification of survival by RYR1 mutation status. Although it shows no significant difference in overall survival time between patients with and without mutations in all cancer types (Fig. 7A), the potential for stratification on prognosis by RYR1 mutations was found in LUAD, adrenocortical carcinoma (ACC), cervical squamous cell carcinoma and endocervical adenocarcinoma (CESC), kidney chromophobe (KICH), kidney renal papillary cell carcinoma (KIRP), acute myeloid leukemia (LAML) and uterine corpus endometrial carcinoma (UCEC) (Fig. 7B–H, P < 0.1). Stratification was insignificant in ACC, KICH, KIRP, LAML and UCEC due to insufficient number of patients with RYR1 mutations or non-significant P value. However, significant stratification was found in LUAD (P = 0.0067, Fig. 7B). This indicates a better prognosis for LUAD patients with RYR1 mutations than those without RYR1 mutations. The opposite result can be found with CESC, in which patients with RYR1 mutations exhibited a potentially worse overall survival than those without mutations (Fig. 7D, P = 0.063).

**Figure 8:**
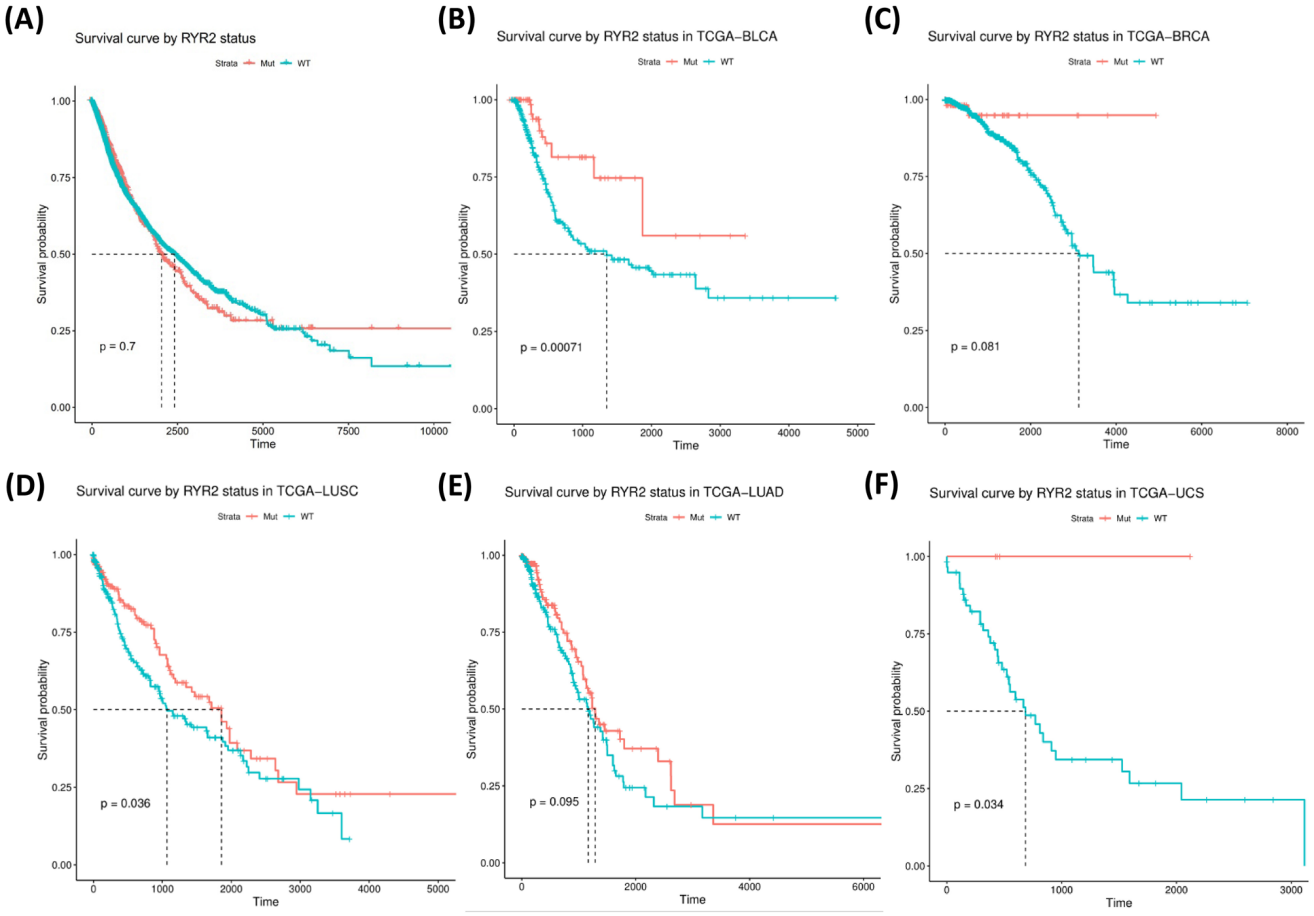
Kaplan–Meier survival analysis based on RYR2 mutational status (mutant or WT). The data for overall survival (OS) time is shown for each subgroup. (**A**) The survival analysis based on RYR2 mutational status for all types of cancers. (**B**–**F**) The survival analysis based on RYR2 mutational status for each type of cancer, as indicated. Only cancer types with P ≤ 0.1 are shown. P values are indicated in each panel. The unit for time is days.

**Figure 9:**
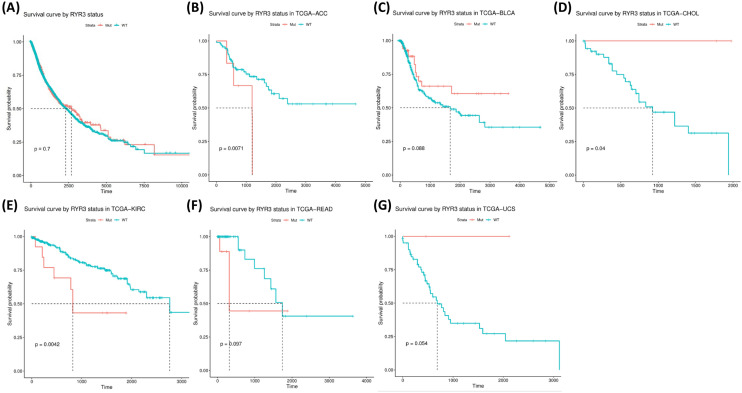
Kaplan–Meier survival analysis based on RYR3 mutational status (mutant or WT). The data for overall survival (OS) time is shown for each subgroup. (**A**) The survival analysis based on RYR3 mutational status for all types of cancers. (**B**–**G**) The survival analysis based on RYR3 mutational status for each type of cancer, as indicated. Only cancer types with P ≤ 0.1 are shown. P values are indicated in each panel. The unit for time is days.

We further analyzed the stratification of prognosis by RYR2 and RYR3 mutations. The results showed that the mutational status of RYR2 did not stratify the survival in all cancer types (Fig. 8A), while significant stratification can be found in bladder urothelial carcinoma (BLCA) (P = 0.00071, Fig. 8B), LUSC (P = 0.036, Fig. 8D) and uterine carcinosarcoma (UCS) (P = 0.034, Fig. 8F). A trend of stratification by RYR2 mutations can also be found in breast invasive carcinoma (BRCA) and LUAD (Fig. 8C,E). Similarly, the mutational status of RYR3 cannot stratify the survival in all cancer types (Fig. 9A), but can stratify the patient survival in ACC (P = 0.0071, Fig. 9B) and KIRC (P = 0.0042, Fig. 9E). A trend of stratification can be found in BLCA, CHOL, READ and UCS by RYR3 mutations (Fig. 9C,D,F,G). The above observations thus indicate that the mutational status of RYR1, RYR2 and RYR3 had no significant stratification on the prognosis of patients when all cancer types were involved, but could stratify the prognosis of certain cancer types.

**Figure 10:**
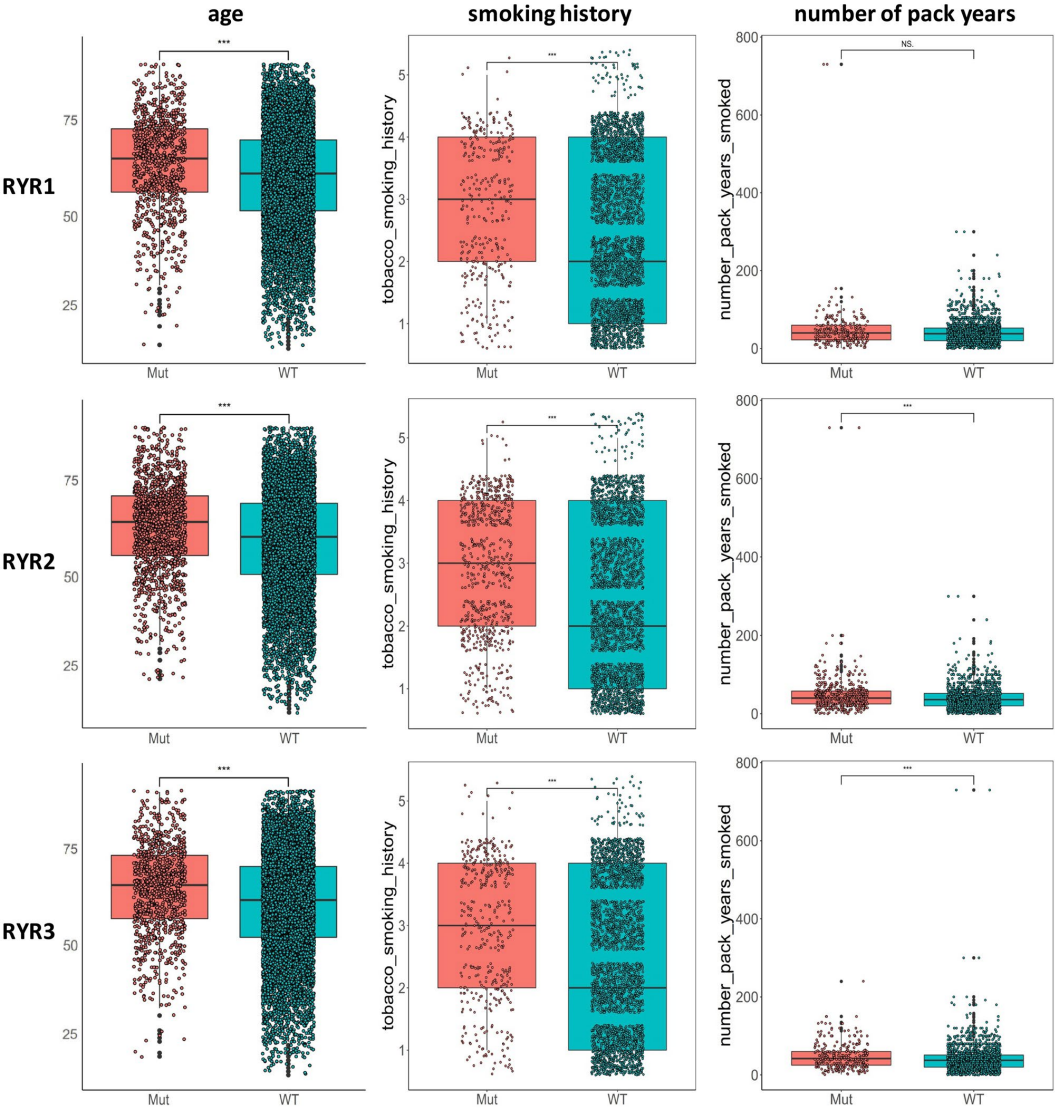
Comparison of age and smoking status between mutant and wild type RYR. Scatter plot of age, smoking history grading and number of cigarette pack years status grouped by RYR1, RYR2 and RYR3 mutational status is shown, as indicated. *P < 0.05; **P < 0.01; ***P < 0.001. Grading of smoking history: 1 = Lifelong Non-smoker (less than 100 cigarettes smoked in Lifetime); 2 = Current smoker (includes daily smokers and non-daily smokers or occasional smokers); 3 = Current reformed smoker for > 15 years (greater than 15 years); 4 = Current reformed smoker for ≤ 15 years (less than or equal to 15 years); 5 = Current reformed smoker, duration not specified.

The relationship between mutational status and age, smoking history, or cigarette consumption, all known cancer risk factors, was also studied. Figure 10 shows that patients with RYR1, RYR2 or RYR3 mutations were significantly older at first diagnosis than those without mutations (P < 0.001). Patients with mutations had significantly higher smoking history scores than those without mutations (P < 0.001), and patients with mutations had significantly higher number of pack years than those without mutations (P < 0.001 for RYR2 and RYR3, not significant for RYR1). Thus, RYR mutational status was highly correlated with patient age, smoking history, and cigarette consumption.

## Discussion

### Characteristic RYR mutational profile reflect pan-cancer genomic alterations in cancers

In this study, we systematically analyzed the mutational status of RYR in more than 30 cancer types, and correlated the mutational status with diagnostic and prognostic factors of patients. We found a random distribution of RYR mutations in cancers with no obvious hot spot mutations. Although the number of mutations in RYR2 was significantly higher than RYR1 and RYR3, the ratio of mutation categories in most cancer types was relatively consistent. The difference between RYR2 and RYR1/3 in the number of mutations may be caused by the differential distribution of different RYR isoforms in various tissues. It is widely knows that RYR1 is the muscular type, RYR2 is the cardiac type while RYR3 is the brain type. However, cancers are rarely seen in muscular and cardiac tissues, therefore, the distribution of RYR in other tissues may not be even. RYR2 may be more expressed than RYR1 and RYR3 in tissues such as lung and gastrointestinal organs and tract, where the incidence of cancers is high, and this may lead to the identification of more mutations predominantly in RYR2 than RYR1 or RYR3.

Patients with RYR mutations had a significantly higher TMB than patients without RYR mutations, suggesting a strong correlation between mutations and TMB. In addition, linear correlation of average number of mutations between RYR isoform pairs indicated consistent mutational status and distribution for all RYR isoforms across different cancer types. In this study, we reported for the first time the stratification capability of RYR mutational status on patient prognosis. Although no stratification was found when all cancers were involved, significant stratification on survival was identified in individual cancer types, including LUAD, LUSC, CESC, BLCA and BRCA, etc. Furthermore, we observed significant associations between mutational status and age or smoking history. In brief, our findings revealed for the first time the full landscape of RYR mutations in cancers and its association with prognosis.

Few previous studies have reported the roles of RYR mutations in cancers. One TCGA database analysis revealed frequent somatic mutations and aberrant promoter methylation and reduced expression of RYR2 in head and neck cancer, suggesting that reduced RYR2 expression in adjacent tissue and precancerous lesions might serve as risk factor for unfavorable prognosis and malignant conversion^5^. There are three possible reasons for the very limited reports on RYR mutations. First, the aberrant Ca^2+^ signaling involves many proteins in a network besides RYRs. Secondly, most of the mutations of RYR in cancers may not substantially affect the channel function. Thirdly, RYR is not among the known tumor driver genes, and its mutations may be the result of carcinogenesis rather than the cause. Our findings suggested that most silent or missense mutations may not significantly compromise RYR protein function, because no key mutations were found in known RYR-related diseases, such as catecholaminergic polymorphic ventricular tachycardia (CPVT) (RYR2) or malignant hyperthermia (RYR1)^6, 7^, indicating that they may not lead to serious phenotypes observed in muscle and heart diseases. In contrast, INDEL and truncation mutations may cause loss of function and impairment of Ca^2+^ signaling, but it may be compensated by other Ca^2+^ signaling mechanisms^8^. Therefore, to what extent RYR loss-of-function mutations affect Ca^2+^ signaling in cancers is largely unknown, and in vitro experiments are needed to examine the functional alterations of these mutations. INDEL and truncation mutations accounted for a very small proportion of mutations (Fig. 2) that happened to limited number of patients, and as these mutations were not hotspot mutations with low mutational frequency, they therefore may not have substantial influences in cancer populations.

Random distribution of mutations generally indicates characteristic cancer mutation landscape in non-driver genes, which has been observed in aging and tumor development as an indication for mutational accumulation^9^. The fact that RYRs had random distribution without hot-spot mutations therefore suggested that most mutations in RYRs may be caused by cancer-related mutational accumulation. Our observations showed that RYR exhibited good linear correlation between the cumulative number of mutations and TMB, suggesting that RYR mutational status reflected whole genome mutational burden. Furthermore, significant differences in TMB between RYR with and without mutations were identified in almost all cancer types, suggesting that mutations in this large protein were correlated with higher TMB. In general, the mutational status of RYRs may be an epitome of the whole genomic alterations.

The type of correlation observed between RYR mutations and TMB may not be unique to RYR, but may also be present in other big proteins. Since RYR mutations appeared to be random, it reflected the genomic mutational load. It can be speculated that similar distribution of mutations may also be present in other big proteins. This was partially proved by the observations in Supplementary Fig. 1, where high mutational frequency can also be observed in VGCCs and IP3 receptors, which are also big proteins in calcium signaling. Furthermore, the differences in the number of mutations and the distinct stratification in different cancers across RYR isoforms may possibly suggest differential distribution of RYRs in various tissues and organs. It is possible that RYRs are differentially affected in different cancers, reflecting distinct roles of RYRs in abnormal Ca^2+^ signaling in different cancers. On the other hand, good correlation of average number of mutations between RYR isoforms suggested similar mutational status in RYRs across various cancers.

Based on current available evidence, we would speculate that RYR mutations are results rather than causes of cancer. This is based on following observations. First, RYRs are not known driver genes of cancer; Secondly, although the mutational frequency of RYR ranked high in some cancers, most mutations appeared to be randomly distributed, possibly due to accumulation of DNA repair mistakes; Thirdly, significant correlation was identified between RYR mutations and TMB; Fourthly, similar mutational pattern and frequency can also be observed in other large proteins of calcium signaling compared with RYR, such as those illustrated in Supplementary Fig. 1.

### The potential roles of aberrant Ca^2+^ signaling in cancers

Aberrant Ca^2+^ signaling has been comprehensively observed in various types of cancers, including lung, colorectal, breast and prostate cancer, etc^1^. The mutational features observed in RYRs may be present in other proteins in Ca^2+^ signaling, especially in those large channels, such as IP3R, SERCA and transient receptor potential channels (TRPCs)^1^. These may lead to Ca^2+^ signal remodeling in cancer, mainly involving expression remodeling and activity remodeling^1^. Expression changes in Ca^2+^ channels and/or pumps can be a common feature across many cancer types. For example, downregulation of SERCA3 during colon carcinogenesis was reported to be an early event in cancer development^10^, and overexpression of TRPM8 was observed in prostate, breast, colon, pancreatic and lung cancers^11, 12^. On the other hand, protein activity remodeling involves activity-regulating proteins, post-translational modifications, splicing and trafficking^13–15^. For example, the proteolytic cleavage of TRPC5^16^ and L-type Ca^2+^ channels^17^ can yield either inactive or more active forms, thus affecting the channel activity.

Aberrant Ca^2+^ signaling may lead to many abnormalities, including inappropriate initiation of transcription, dysregulated kinase activation and inactivation, altered epigenetic processes such as methylation and hydroxymethylation and aberrant inflammation pathways (such as NF-Kappa B)^18^. These aberrancies can be caused by cancer microenvironment change, epigenetic and genetic changes, and can further alter the microenvironment, genetic and epigenetic regulation^18^.

### Correlation of cancer prognostic factors involving RYR mutational status

In this study, we found that the survival of patients in several cancer types was significantly stratified by RYR mutational status, but exhibited differential stratification in two aspects. First, better survival was observed in patients with mutations or without mutations in different cancers. For patients with lung cancer (LUAD and LUSC) or BLCA, those with RYR mutations exhibited better overall survival than those without mutations. In contrast, for patient with CESC and kidney cancers (KICH, KIRP and KIRC), those without mutations exhibited better overall survival than those with mutations. Secondly, RYR isoform specific stratification can be observed. For example, significant LUAD stratification can be found in RYR1 but not in RYR3, and significant LUSC stratification can be found in RYR2 but not in RYR1 and RYR3. The reasons for the differential stratification could include the organ-specific RYR isoform distribution and the altered RYR expression or activity during carcinogenesis. It is wildly accepted that RYR1 is the skeletal muscle type, RYR2 is the cardiac muscle type and RYR3 is found at lower levels throughout many tissues including the brain^4^. However, the profile of RYR expression and distribution in specific human cancer tissues is largely unknown. We suppose that specific RYR isoforms could be highly expressed in cancer tissues where large amount of RYR mutations were found. For example, RYR1 and RYR2 could be highly expressed in LUAD tissues, and RYR2 and RYR3 could be highly expressed in BLCA tissues. Thirdly, cancer generally happens to epithelial tissues, whether they are adenocarcinoma or squamous cell carcinoma, with the most common cancers including lung adenocarcinoma, lung squamous cell carcinoma, bladder squamous cell carcinoma, cervical squamous cell carcinoma, endometrioid adenocarcinoma, colorectal adenocarcinoma and gastric adenocarcinoma. Therefore, the distribution of RYR in epithelial tissues is important in defining the RYR mutational status in these cancers. It appeared that RYRs, especially RYR2 may be highly expressed in epithelial cells.

High nonsynonymous TMB was shown to be associated with a better prognosis in patients with resected NSCLC, suggesting that TMB was an independent predictor for NSCLC prognosis^19^. We found that RYR mutations exhibited high correlation with somatic TMB, suggesting that the potential of RYR mutations in prognosis stratification may be derived from and similar to TMB. TMB is defined as the mutational burden of the whole exome while RYR only represents a small segment of exome. However, it appeared from our observation that the small segment may exhibit similar prediction capability, which facilitates its use in future prognosis prediction. TMB is a widely accepted marker for response stratification in immunotherapy. Whether RYR or Ca^2+^ signaling can be a new marker for prognosis prediction and therapeutic response prediction is worth more investigation.

We observed significant correlation between mutational status and smoking status or age. Smoking is a widely-accepted independent risk factor for cancers. It has been shown that smoking was correlated with higher mutational burden in many types of cancers^20, 21^. It was unsurprising that patients with RYR mutations exhibited higher grade of smoking history and higher number of pack years than those without RYR mutations in our study. Our analysis on RYRs again proved that smoking was correlated with higher number of genomic mutations, which may facilitate the malignant transformation of tissues. It has long been known that somatic mutations gradually accumulate with age, and older people may have more background mutations than younger individuals^9^. This was also reflected in our observation with RYR, in which patients with RYR mutations had higher age than those without RYR mutations. Malignant transformation of tissues during carcinogenesis may further increase the amount of mutations over background mutations.

This study had some limitations. First, the relationship between RYR and cancer and its significance in cancer prognosis still need further investigation, as this study represented a preliminary study correlating calcium channel such as RYR with cancer phenotypes. Although RYR mutations were found to be a potential substitution of TMB for prognosis stratification, more evidence and validation are still needed to make it an applicable marker. Secondly, investigation on the potential of RYR mutational status as a prognostic indicator for individual cancer patient is required, since current study only revealed its significance at population level. Thirdly, the mutational status of RYR and its correlation with prognosis for individual cancers may be intriguing, since RYR mutational landscape may vary greatly across different cancer types. Fourthly, deeper understanding and more studies are needed on the roles of RYR and calcium signaling in carcinogenesis and cancer development.

## Supplementary Information

Supplementary Figure 1.

## Data Availability

The datasets generated and/or analyzed during the current study are available and can be downloaded from the TCGA database (https://portal.gdc.cancer.gov/).
