# Supplementary material for: The ryanodine receptor mutational characteristics and its indication for cancer prognosis
Source: Sci Rep. 2022 Sep 27;12:16113. doi: 10.1038/s41598-022-19905-y (PMC9515073; doi:10.1038/s41598-022-19905-y)
Supplement: Supplementary file 1 — Supplementary Figure 1. [file 41598_2022_19905_MOESM1_ESM.pdf]

The ryanodine receptor mutational characteristics and its indication for cancer prognosis

--RYR mutations predict cancer prognosis

Fenglin Wang[1,#], Jingbo Yu[4,#], Ping Lin[5,#], Charalampos Sigalas[2], Shibo Zhang[6], Yuan Gong[7], Rebecca Sitsapesan[2,*], Lele Song[3,*]

1. College of Life Sciences, Nankai University, Tianjin 300071, P.R. China.
2. Department of Pharmacology, University of Oxford, Mansfield Road, Oxford OX1 3QT, UK.
3. Department of Radiotherapy, the Eighth Medical Center of the Chinese PLA General Hospital, Beijing 100091, P.R. China.
4. Department of Hepatobiliary Surgery, Dalian Municipal Central Hospital, Dalian Medical University, Dalian 116033, Liaoning Province, P.R. China.
5. Department of Ultrasound, the First Affiliated Hospital of Dalian Medical University, Dalian 116011, Liaoning Province, P.R. China.
6. Department of Thoracic Surgery, Harbin Medical University Cancer Hospital, Harbin 150081, Heilongjiang Province, P.R. China.
7. Department of Gastroenterology, the Second Medical Center of the Chinese PLA General Hospital, Beijing 100853, P.R. China.

*Corresponding authors:
Dr. Lele Song, The Eighth Medical Center of the Chinese PLA General Hospital, Beijing 100091, P.R.China. Email: songlele@sina.com; Tel: 86-13240149188.
Prof. Rebecca Sitsapesan, Department of Pharmacology, University of Oxford, Mansfield Road, Oxford, OX1 3QT, UK. Email: rebecca.sitsapesan@pharm.ox.ac.uk.

# These authors contributed equally to this study.

Supplementary information

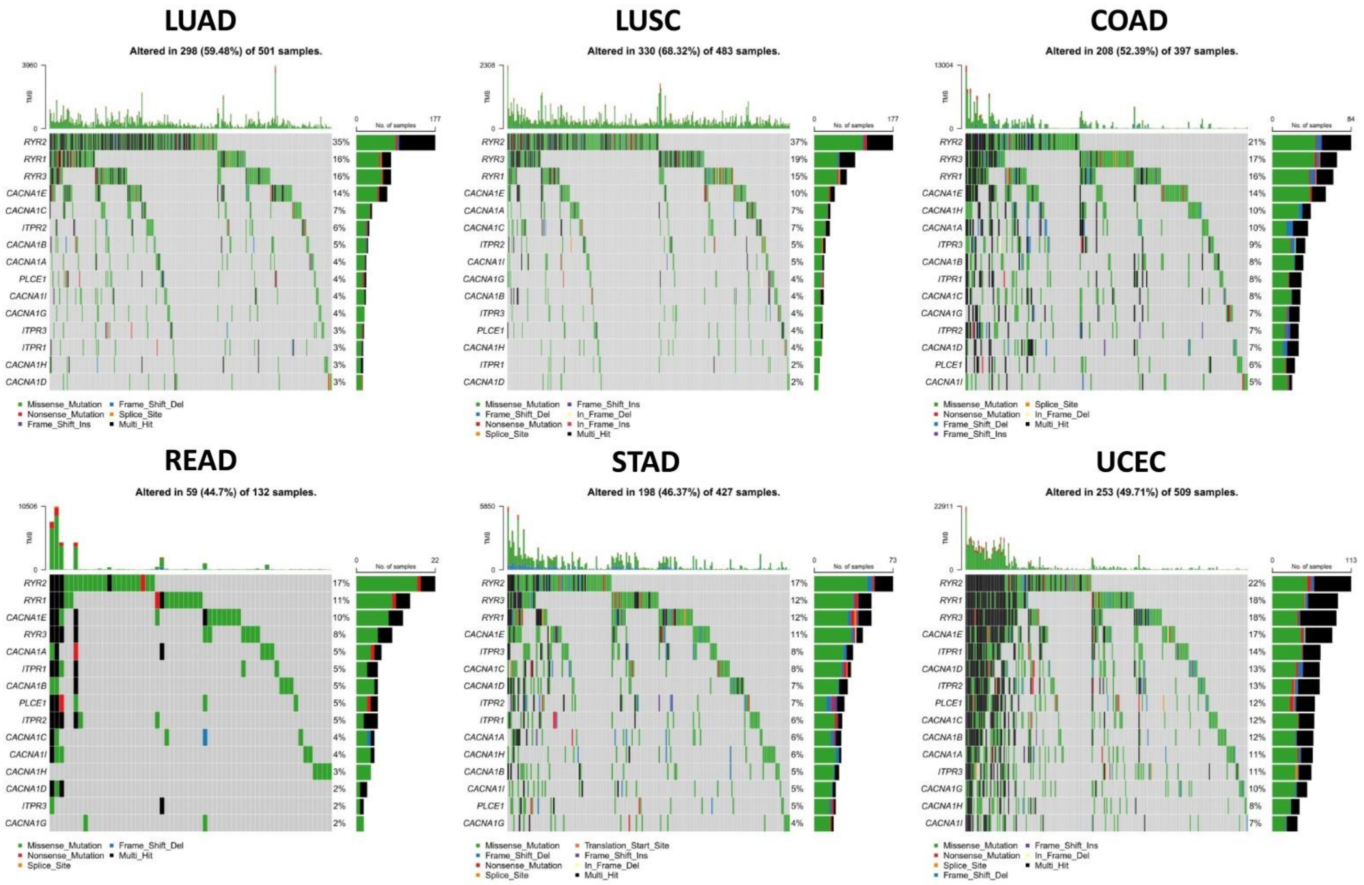

Supplementary Figure 1. The mutational frequency of the top 15 mutated genes in calcium signaling in six representative cancers. The mutational frequency is shown for lung adenocarcinoma (LUAD), lung squamous cell carcinoma (LUSC), colon adenocarcinoma (COAD), rectum adenocarcinoma (READ), stomach adenocarcinoma (STAD) and uterine corpus endometrial carcinoma (UCEC). The top mutated genes mainly included calcium channels, including ryanodine receptor (RYR), voltage-gated calcium channel alpha1 subunit (CACNA1) and inositol 1,4,5-trisphosphate receptor (ITPR), which are crucial membrane proteins of calcium signaling. RYR, especially RYR2, ranked the top in these representative cancers.
